# Supplementary material for: Spatiotemporal-resolved protein networks profiling with photoactivation dependent proximity labeling
Source: Nat Commun. 2022 Aug 20;13:4906. doi: 10.1038/s41467-022-32689-z (PMC9392063; doi:10.1038/s41467-022-32689-z)
Supplement: Supplementary file 1 — Supplementary Information [file 41467_2022_32689_MOESM1_ESM.pdf]

Supplementary Information

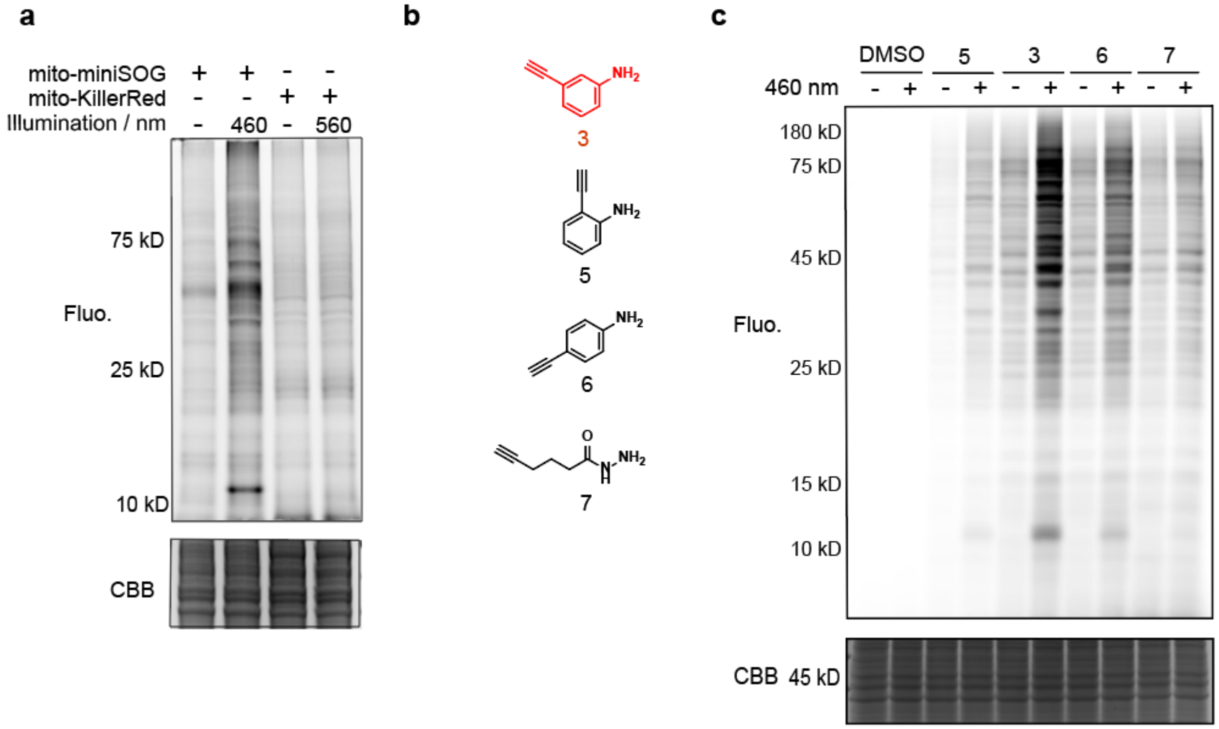

Supplementary Figure 1 | Development of the PDPL approach. **a)** HEK293T cells stably expressing miniSOG and KillerRed in mitochondria were tested in the presence or absence of their corresponding excitation wavelength. Propargyl amine (probe **2**) was used in this experiment. **b)** The chemical structure of amine probes **5-7**. **c)** Representative fluorescent gel analysis of mitochondria-localized miniSOG mediated proteomic labeling with probes **5-7** and probe **3** as the internal control. Negative control experiments omitting blue light or without probe treatment were used to evaluate the signal-to-background labeling of the chemical probes. These experiments were independently repeated at least twice with similar results.

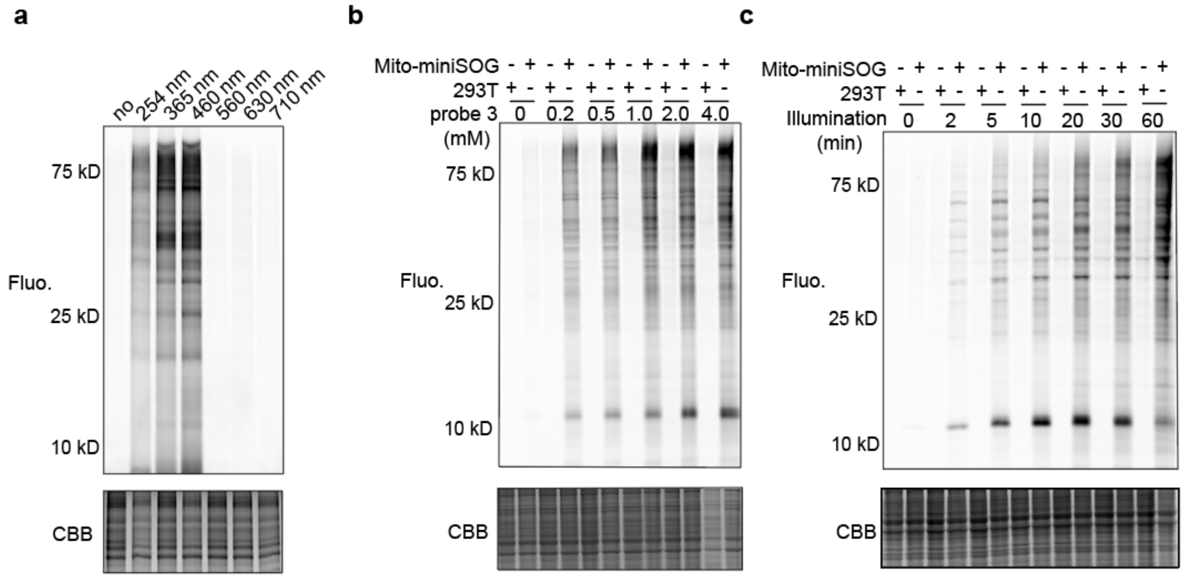

Supplementary Figure 2 | Optimization of PDPL conditions. **a)** Different wavelength of illumination was tested in HEK293T-miniSOG-mito in the presence of optimized probe **3**. **b)** The concentrations of optimized probe **3** were tested in HEK293T-miniSOG-mito as the experimental group and normal HEK293T as the control. 1 mM probe reached to labeling plateau and was selected. **c)** The illumination duration was screened similar to **b**. 20 min was selected as the optimized condition. CBB: coomassie brilliant blue. These experiments were independently repeated at least twice with similar results.

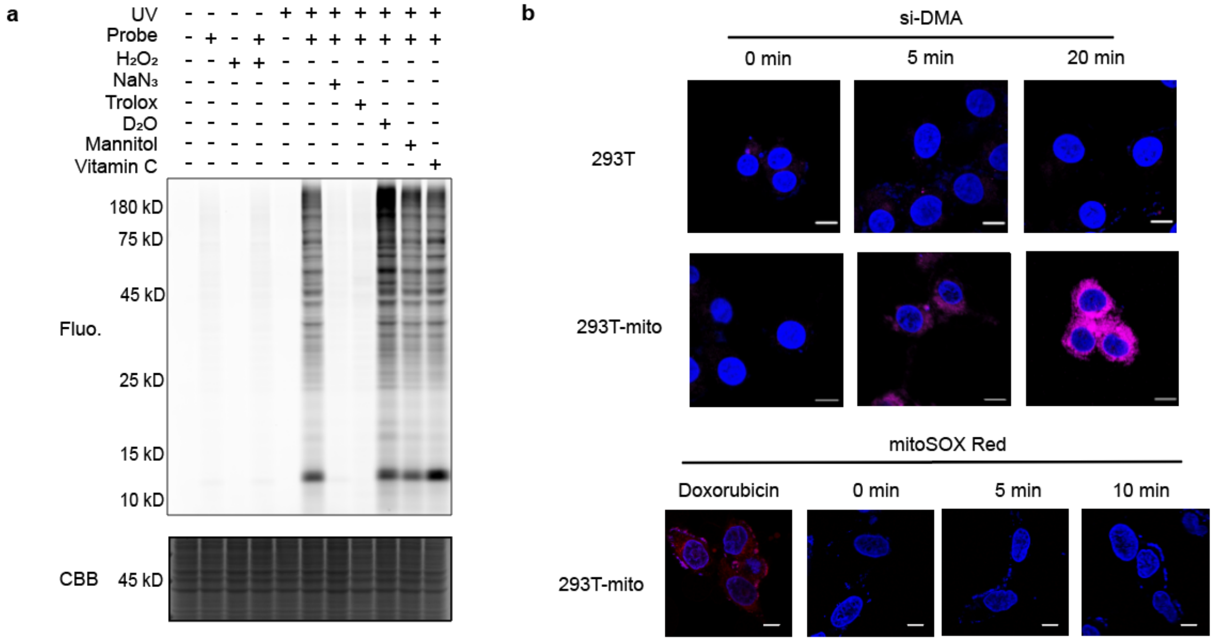

Supplementary Figure 3 | a) Investigation of the ROS type generated in PDPL. Trolox and sodium azide could quench the labeling, while D_2O could enhance the labeling. PDPL was insensitive to Mannitol and Vitamin C and H_2O_2 cannot trigger the labeling. **b)** Fluorescent imaging of singlet oxygen by Si-DMA

probe confirmed the presence of singlet oxygen in HEK293T-miniSOG line but not parental HEK293T line. mitoSOX Red was unable to detect the superoxide after illumination. Doxorubicin was used as a positive control. Scale bar: 10 μm. These experiments were independently repeated at least twice with similar results.

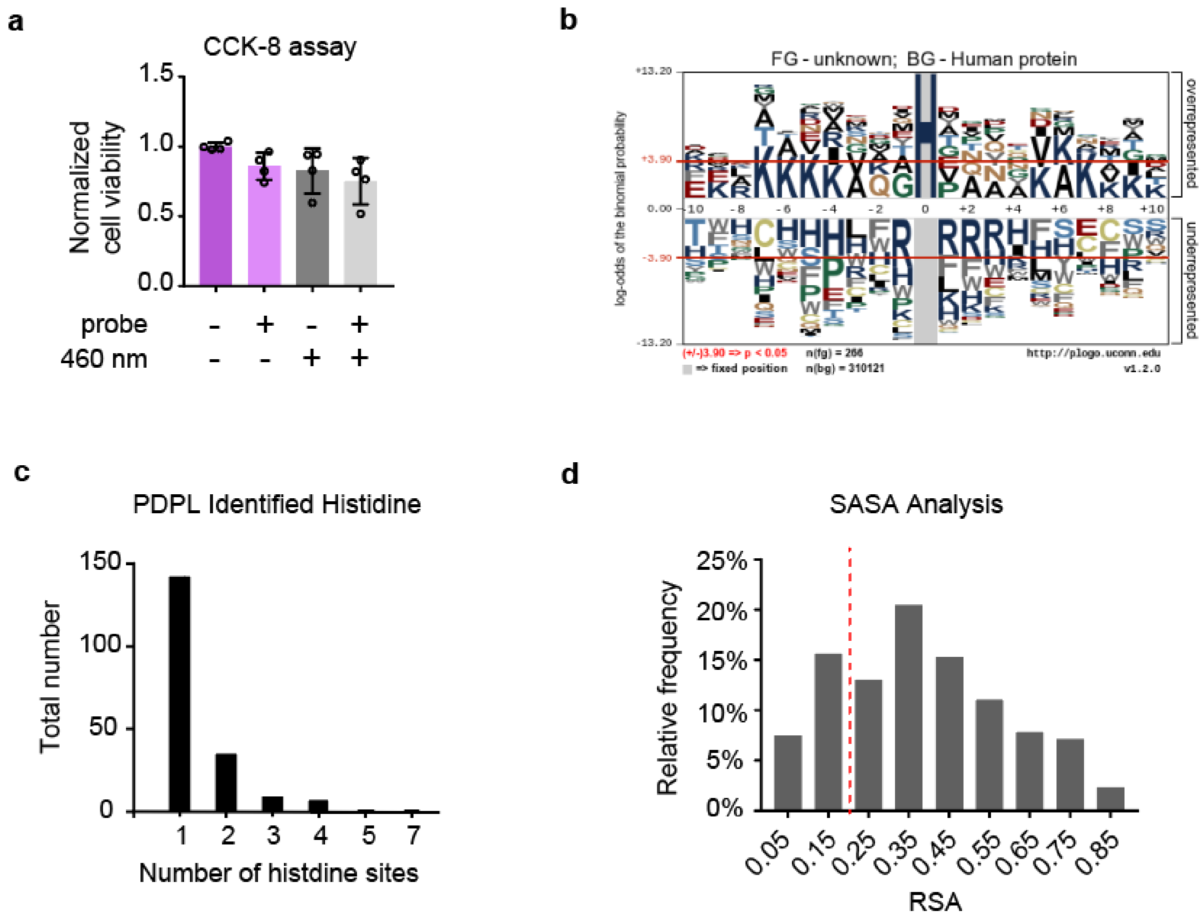

Supplementary Figure 4 | a) Evaluation of cell toxicity of PDPL. CCK-8 assay was deployed to measure the cell viability under shown conditions. n = 4 biologically independent samples. Data are presented as mean values +/- SD. **b)** pLOGO analysis[1] of the local sequence context of PDPL-modified histidine revealed a modest motif preference for small, hydrophobic residues at the ±1 position. **c)** Histogram plot of PDPL identified histidines per protein. **d)** Relative solvent accessibility (RSA) analysis of the identified histidine. The routinely adopted threshold 20% were labeled.

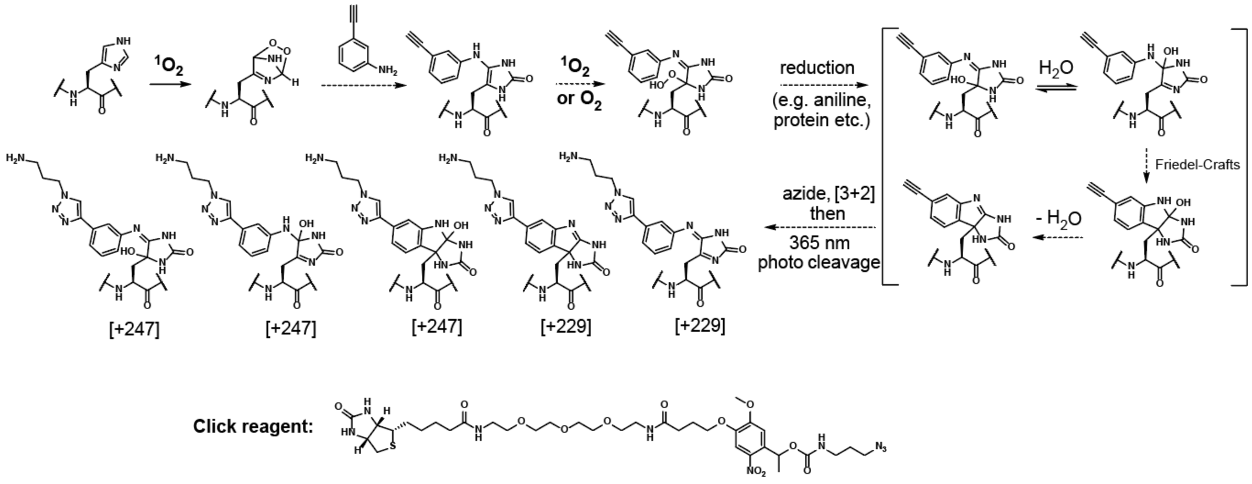

Supplementary Figure 5 | Proposed chemical mechanism for PDPL. According to published mechanism of histidine oxidation by singlet oxygen[2, 3], probe **3** underwent addition to 2-oxo-histidine followed by secondary oxidation process similar to those of FAD-dependent monooxygenase[4]. The intermediate underwent reduction in aqueous solution and intramolecular Friedel-Crafts reaction may occur afterwards[5, 6]. After click chemistry and photo cleavage, final products with delta masses +229 and +247 were generated.

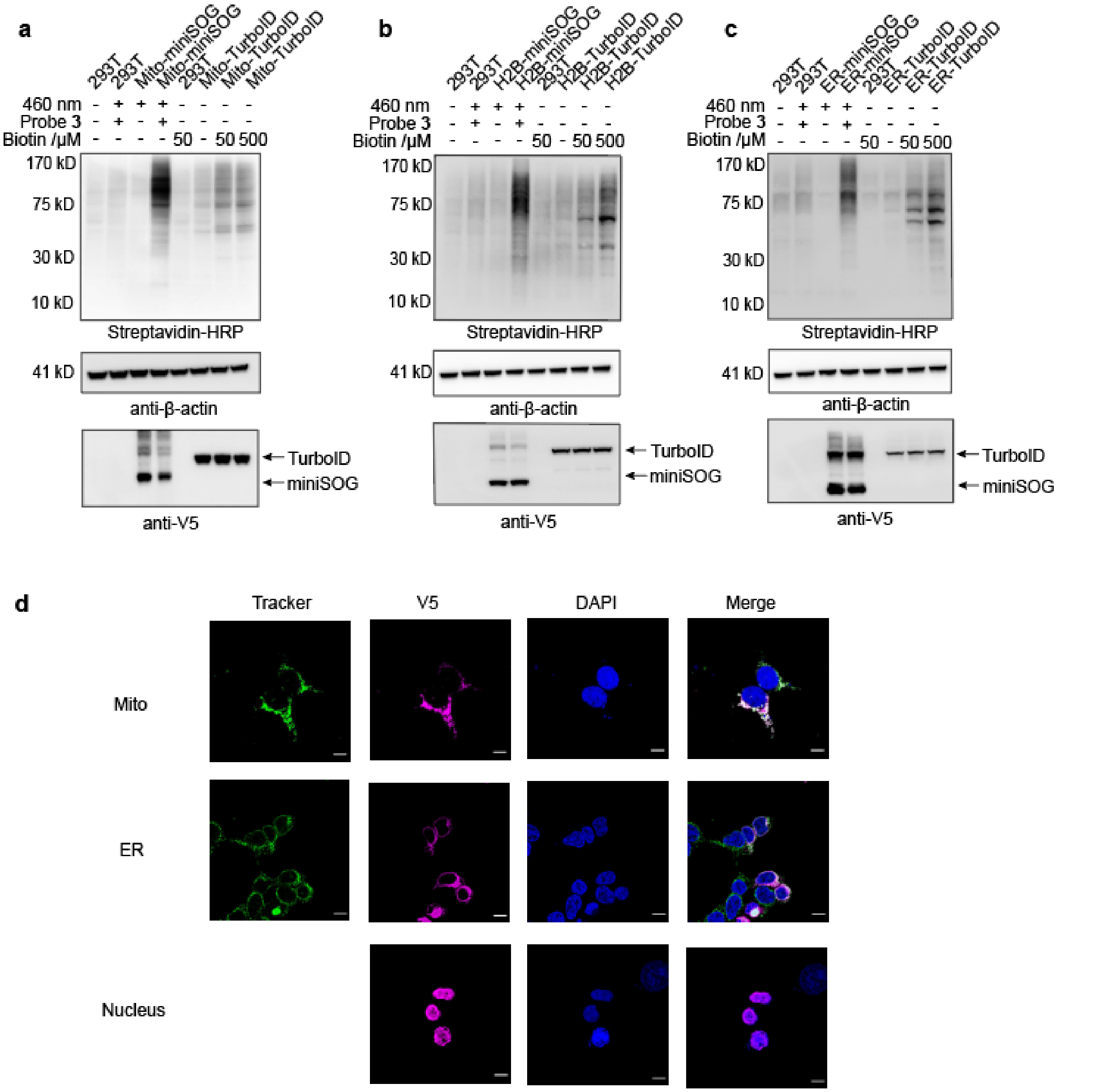

Supplementary Figure 6 | Side-by-side comparison of PDPL to TurboID in sub-organelle protein profiling. **a-c)** Representative gel imaging of three organelle-specific labeling comparisons of PDPL to TurboID. miniSOG and TurboID were located in mitochondria, nucleus and ER. 50 μM and 500 μM exogenous biotin were added in TurboID experiments. β-actin was used as the protein loading control and V5 tag was used to show similar levels of miniSOG and TurboID are being expressed. **d)** Fluorescent imaging of the TurboID constructs to show the correct localizations for three organelles. Scale bar: 10 μm. These experiments were independently repeated at least twice with similar results.

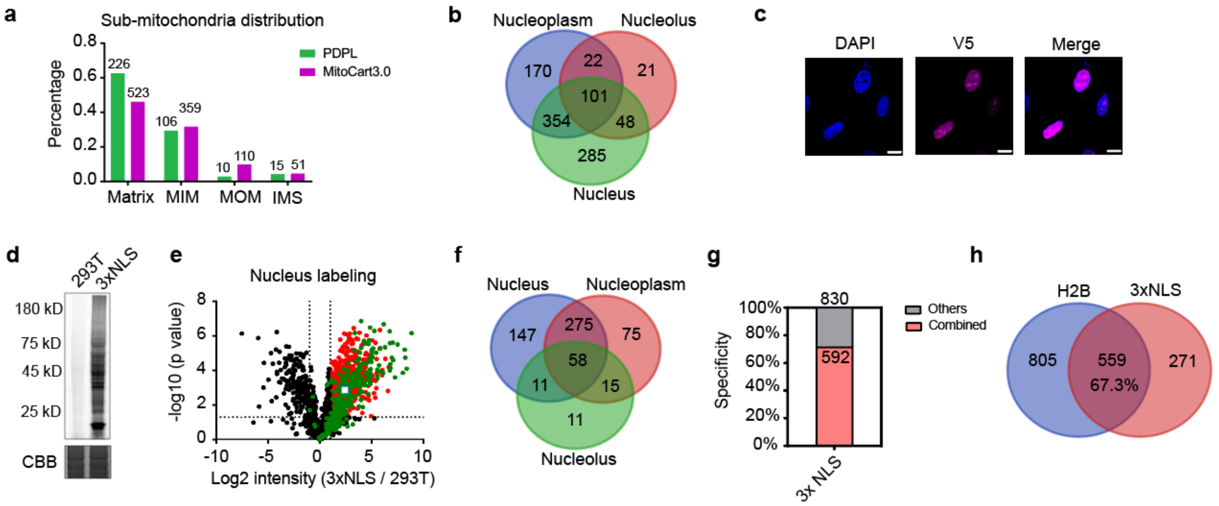

Supplementary Figure 7 | a) Submitochondrial analysis of the PDPL identified mitochondrial proteins as well as the whole dataset collected in the MitoCart3.0 database. Matrix: mitochondrial matrix; MIM: mitochondrial inner membrane; MOM: mitochondrial outer membrane; IMS: intermembrane space. The protein number identified in each sub-organelle were listed on the top. **b)** Subnuclear analysis of the PDPL identified real nucleus proteins by miniSOG-H2B construct. Venn diagram showed the overlapping proteins in nucleoplasm, nucleus and nucleolus. **c)** Fluorescent imaging of miniSOG-3xNLS construct for its nucleus localization. Scale bar: 10 μm. **d)** Gel imaging of nucleus specific PDPL labeling by miniSOG-3xNLS. **e)** Volcano plots of PDPL-labeled proteome by miniSOG-3xNLS using label free quantification (n = 3 independent biological experiments). Two-sided student's t-test was used in the volcano plot. Significantly changed proteins are highlighted in red ($p < 0.05$ and >2-fold ion intensity difference). Relevant proteins that are significant in HEK293T-miniSOG but not in HEK293T are marked in green. **f)** Subnuclear analysis of the PDPL identified real nucleus proteins by miniSOG-3xNLS construct. Venn diagram showed the overlapping proteins in nucleoplasm, nucleus and nucleolus. **g)** Specificity analysis for miniSOG-3xNLS derived from experiment in **e**. **h)** Venn diagram showing overlapping proteins in miniSOG-H2B and miniSOG-3xNLS constructs. These experiments were independently repeated at least twice with similar results.

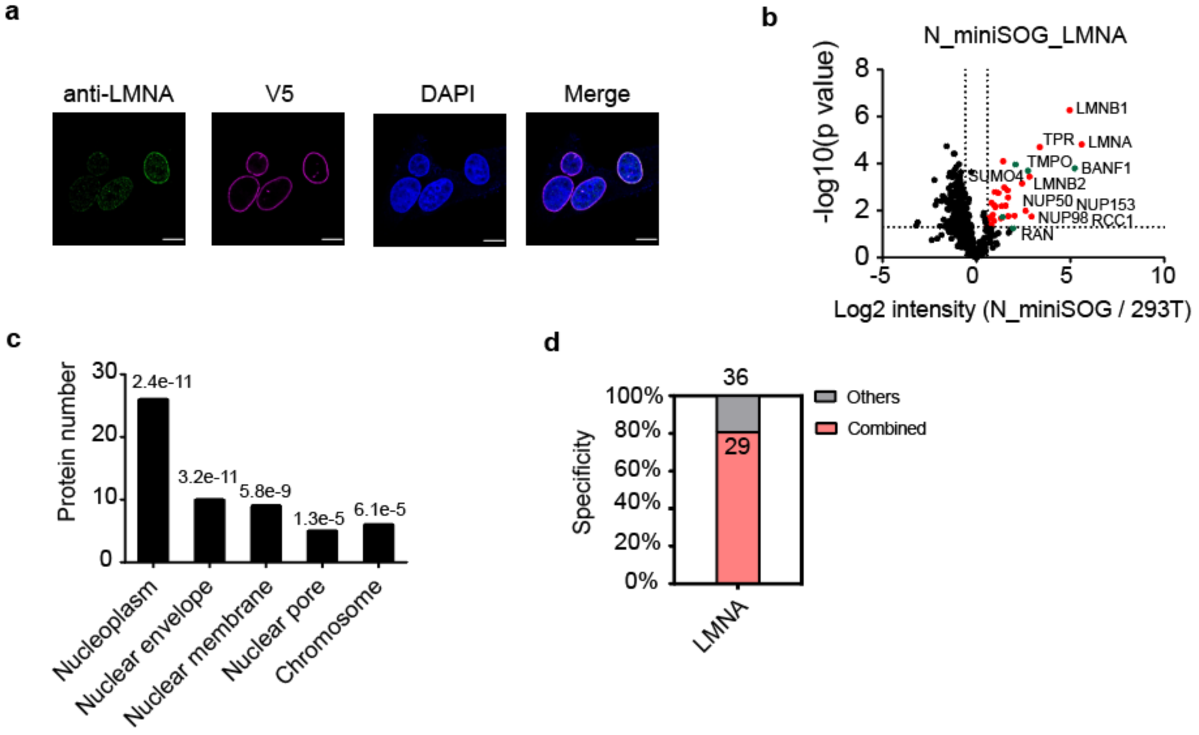

Supplementary Figure 8 | a) Confocal imaging of HEK293T cells expressing miniSOG-Lamin A which were detected with Lamin A antibody, V5 tag antibody and DAPI. Scale bar: 10 µm. **b)** Volcano plot of PDPL-labeled proteome in miniSOG-Lamin A expressing cells by label free quantification ($n = 3$ independent biological experiments). Two-sided student's t-test was used in the volcano plot. Significantly changed proteins are highlighted in red ($p < 0.05$ and >1.5-fold ion intensity difference). Relevant proteins that are significant in HEK293T-miniSOG but not in HEK293T are marked in green. **c)** GO analysis of cellular component revealed the subnuclear localization of the enriched proteins. P values from two-sided student's t-test were labeled on each bar plot. **d)** Specificity analysis for proteomic data set derived from experiments in **b**. Total number of statistically significant proteins was labeled on top. Bar plot shows the combined protein number in **c**. These experiments were independently repeated twice with similar results.

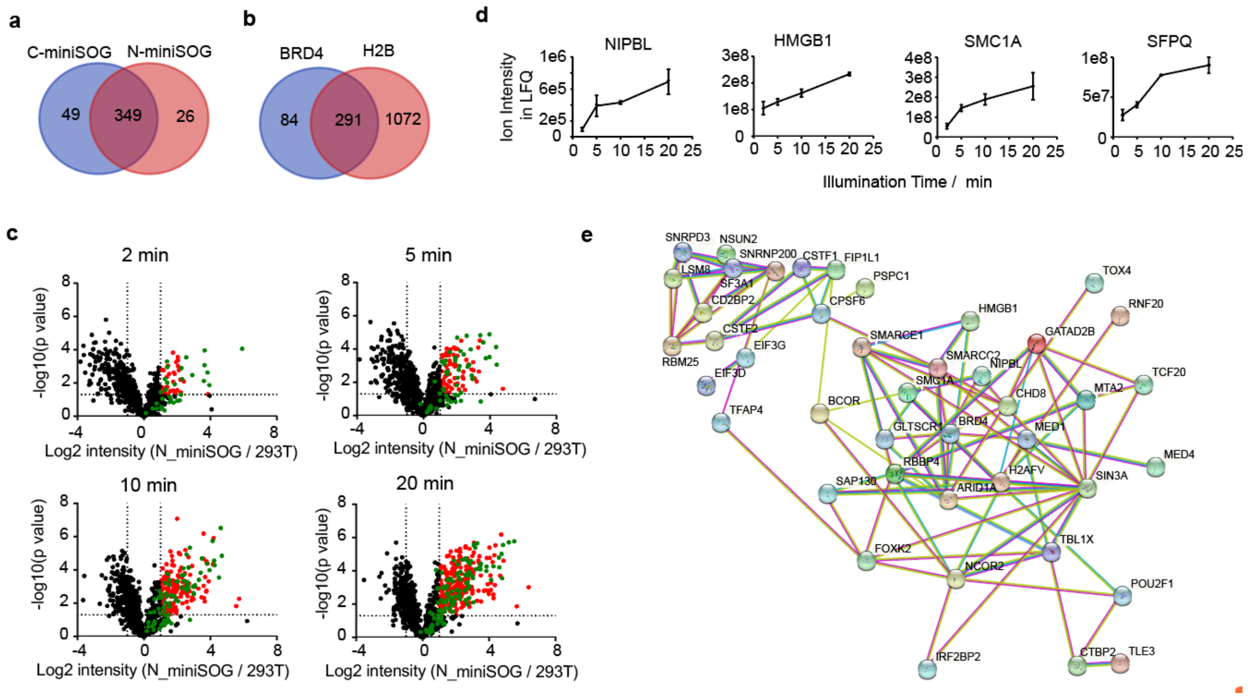

Supplementary Figure 9 | a) Venn diagram showing overlapping proteins in C terminal and N terminal miniSOG fused BRD4. **b)** Venn diagram showing overlapping proteins in miniSOG-BRD4 and miniSOG-H2B. **c)** Volcano plots of PDPL-labeled proteomes with irradiation time: 2 min, 5 min, 10 min and 20 min (n = 3 independent biological experiments). Two-sided student's t-test was used in the volcano plot. HEK293T was used as the negative control. Significantly changed proteins are highlighted in red ($p < 0.05$ and >2-fold ion intensity difference). Relevant proteins that are significant in HEK293T-miniSOG but not in HEK293T are marked in green. **d)** The ion intensity in label free quantification for known BRD4 binding proteins across the indicated irradiation time. n = 3 biologically independent samples. Data are presented as mean values +/- SD. **e)** Full map for string analysis of BRD4 interacting proteins with over three interactors.

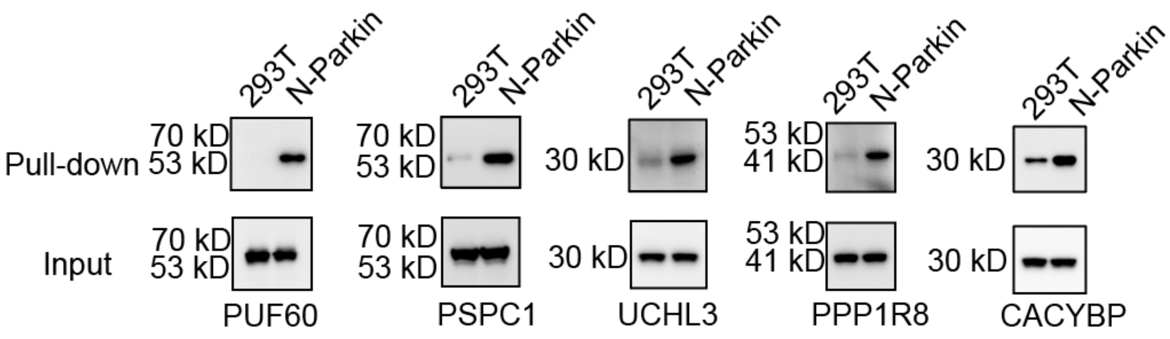

Supplementary Figure 10 | The non-substrate proteins were validated as binding proteins of Parkin. PUF60, PSPC1, UCHL3, PPP1R8 and CACYBP were transfected to HEK293T and HEK293T stably expressing miniSOG. PDPL processing with anti-Flag Western blot was used for detection. The results were consistent with LC-MS/MS. These experiments were independently repeated at least twice with similar results.

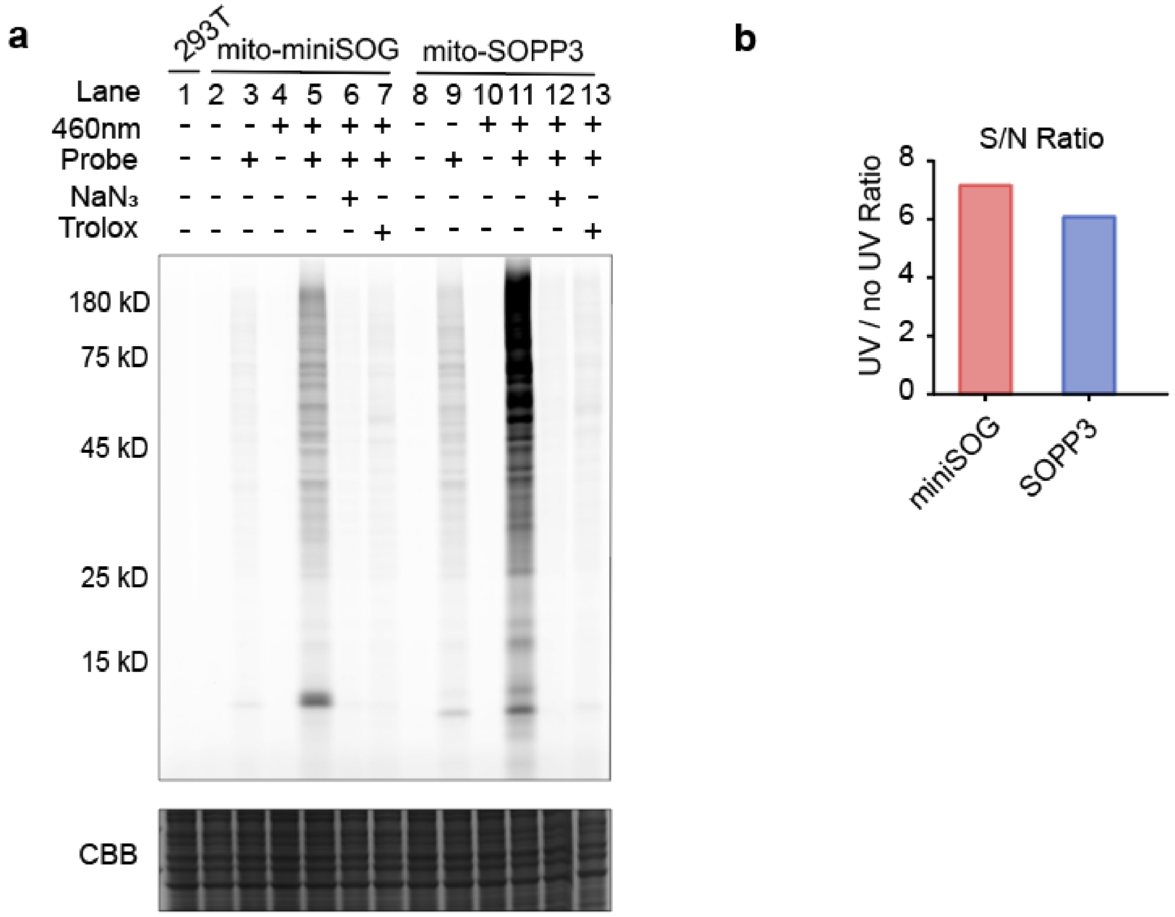

Supplementary Figure 11 | a) Comparison of mito-miniSOG to mito-SOPP3. Both labelings could be quenched by Trolox and NaN_3. Omission of illumination or aniline probe was tested for specificity. CBB: coomassie brilliant blue. **b**) signal-to-noise ratio was evaluated based on illumination and no illumination for both proteins (miniSOG: lane 5 / lane 3; SOPP3: lane 11 / lane 9). These experiments were independently repeated at least twice with similar results.

Supplementary Table 1 | Genetic constructs used in this study

Name	Vector	Location	Features
mito-miniSOG	pLX304	Mitochondrial matrix	BstBI-mito-V5-miniSOG-NheI
mito-SOPP3	pLX304	Mitochondrial matrix	BstBI-mito-V5-SOPP3-NheI
miniSOG-Sec61β	pLX304	ER mrmbrane (ERM)	BstBI-V5-miniSOG-Sec61β-NheI
miniSOG-H2B	pLX304	Nucleus	BstBI-V5-miniSOG-H2B-NheI
miniSOG-3xNLS	pLX304	Nucleus	BstBI-V5-miniSOG-3xNLS-NheI
mito-TurboID	pLX304	Mitochondrial matrix	BstBI-mito-V5-TurboID-NheI
TurboID-Sec61β	pLX304	ER mrmbrane (ERM)	BstBI-V5-TurboID-Sec61β-NheI
TurboID-H2B	pLX304	Nucleus	BstBI-V5-TurboID-H2B-NheI
**Name**	**Vector**	**Features**	
miniSOG	pET21a	BamHI-miniSOG-XhoI-6X His-tag	
minoSOG-BRD4	pLX304	BstBI-V5-miniSOG-G4S-BRD4-NheI	
BRD4-miniSOG	pLX304	BstBI-BRD4-G3S-V5-miniSOG-NheI	
miniSOG-Pakrin	pLX304	BstBI-V5-miniSOG-G4S-Parkin-NheI	
Pakrin-miniSOG	pLX304	BstBI-Parkin-G3S-V5-miniSOG-NheI	
miniSOG-LaminA	pLX304	BstBI-V5-miniSOG-G4S-LaminA-NheI	
SSU72	pLenti	BamHI-SSU72-3xFlag	
SNW1	pLenti	BamHI-SNW1-3xFlag	
UCHL3	pLenti	BamHI-UCHL3-3xFlag	
PPP1R8	pLenti	BamHI-PPP1R8-3xFlag	
CACYBP	pLenti	BamHI-CACYBP-3xFlag	
PUF60	pLenti	BamHI-PUF60-3xFlag	
PSPC1	pLenti	BamHI-PSPC1-3xFlag	
PRDX1	pLenti	BamHI-PRDX1-3xFlag	
PRDX1(H-A)	pLenti	BamHI-PRDX1(H10A, H81A, H169A)-3xFlag	
PRDX3	pLenti	BamHI-PRDX3-3xFlag	
PRDX3(H-A)）	pLenti	BamHI-PRDX3(H155A, H225A)-3xFlag	

Full scans of images:

Supplementary Figure 1

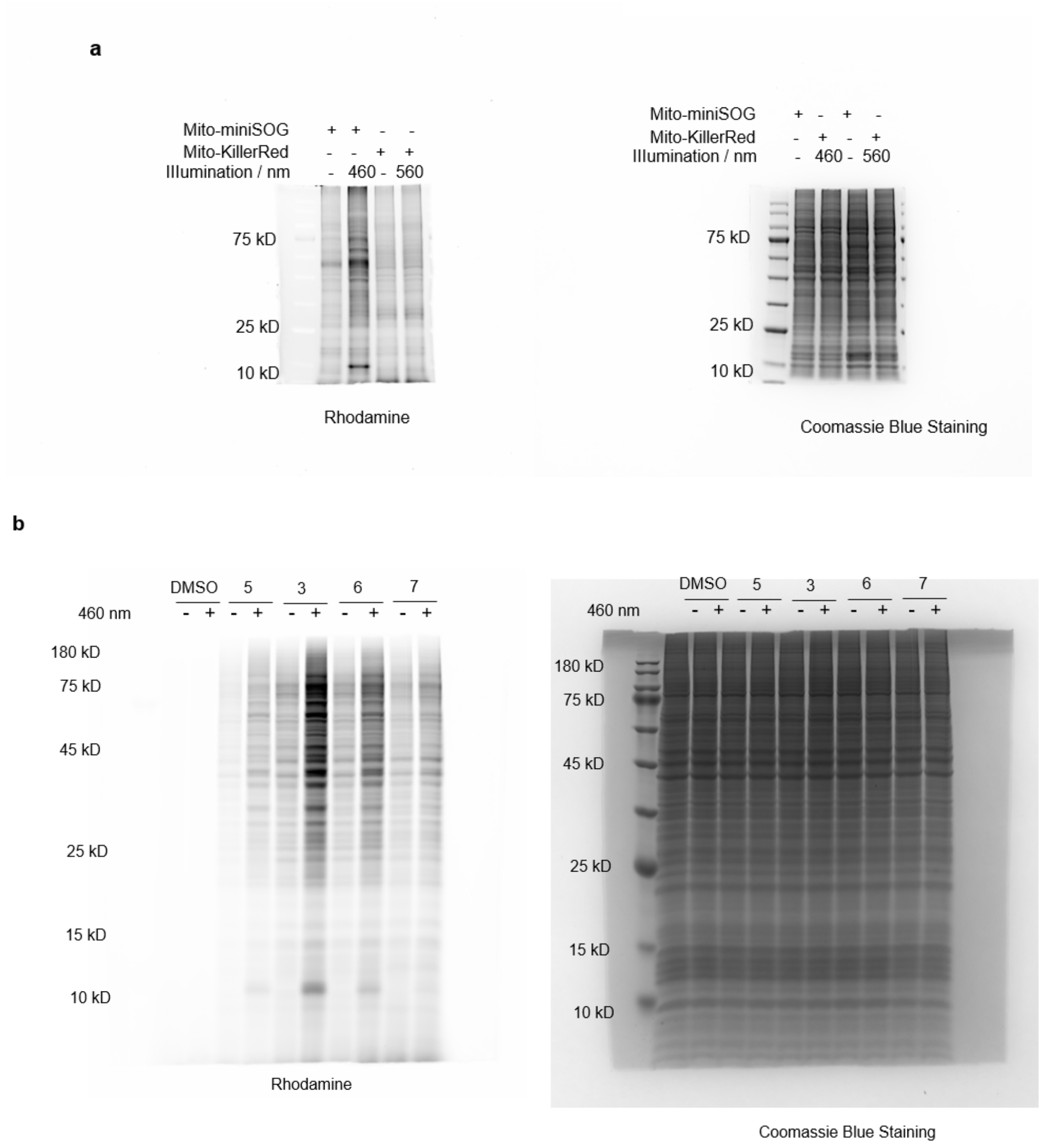

Supplementary Figure 2

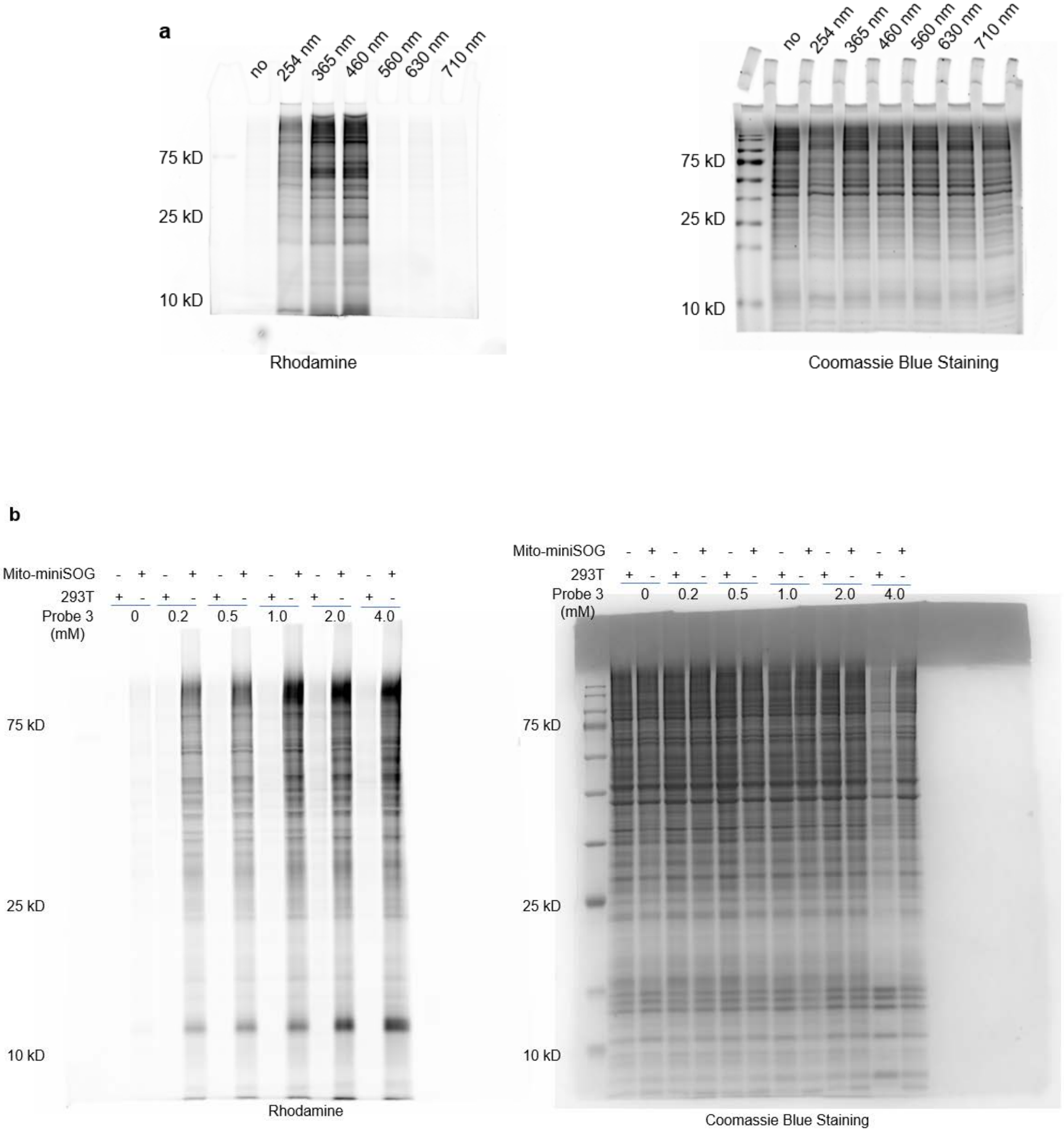

c

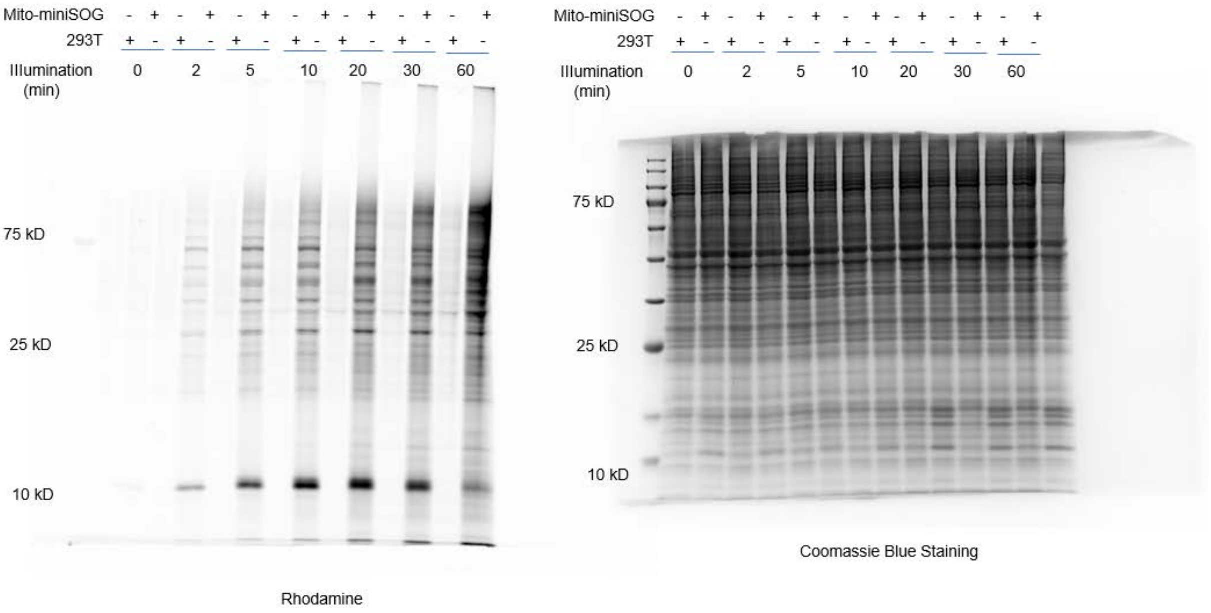

Supplementary Figure 3

a

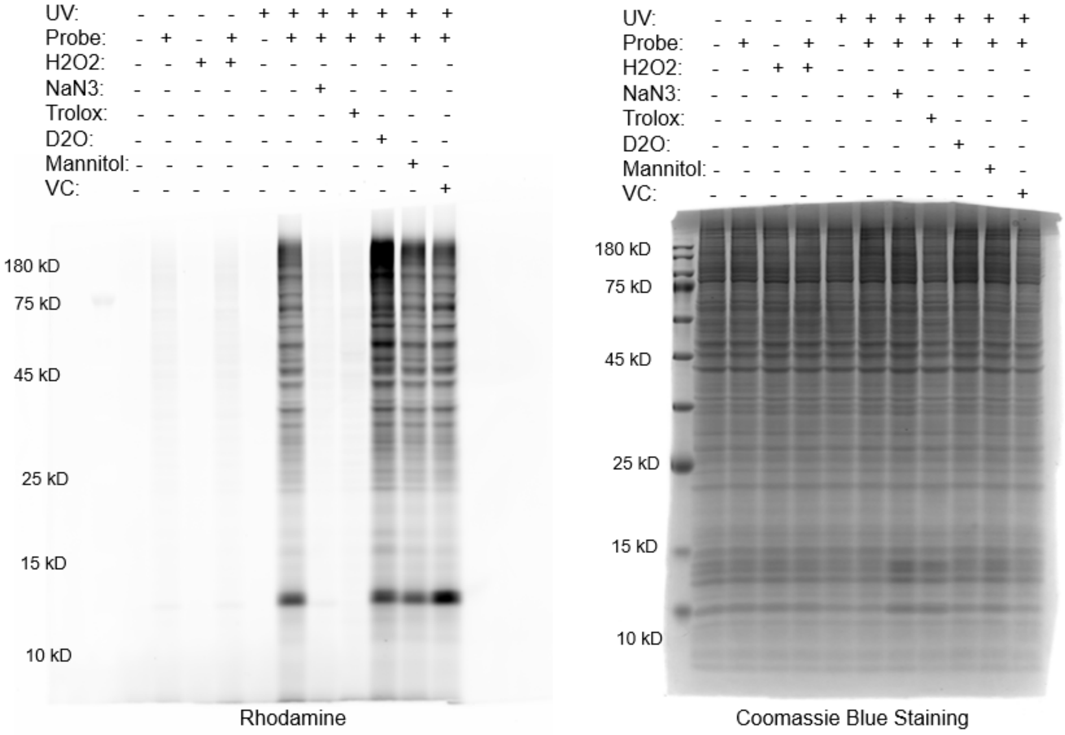

Supplementary Figure 6

a

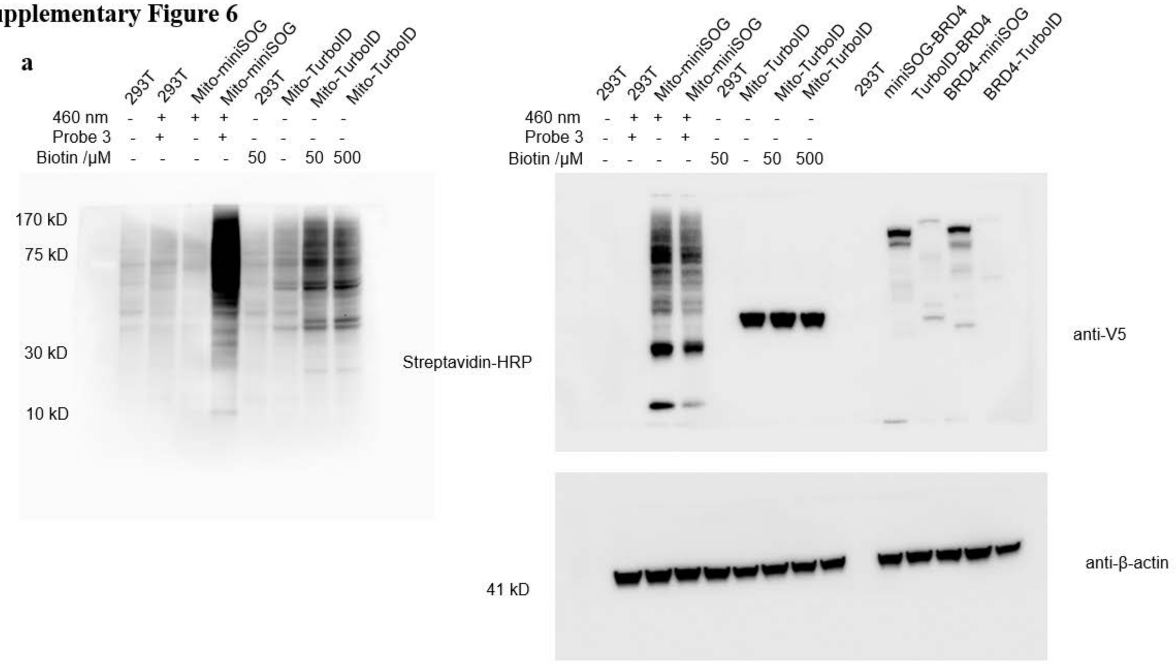

b

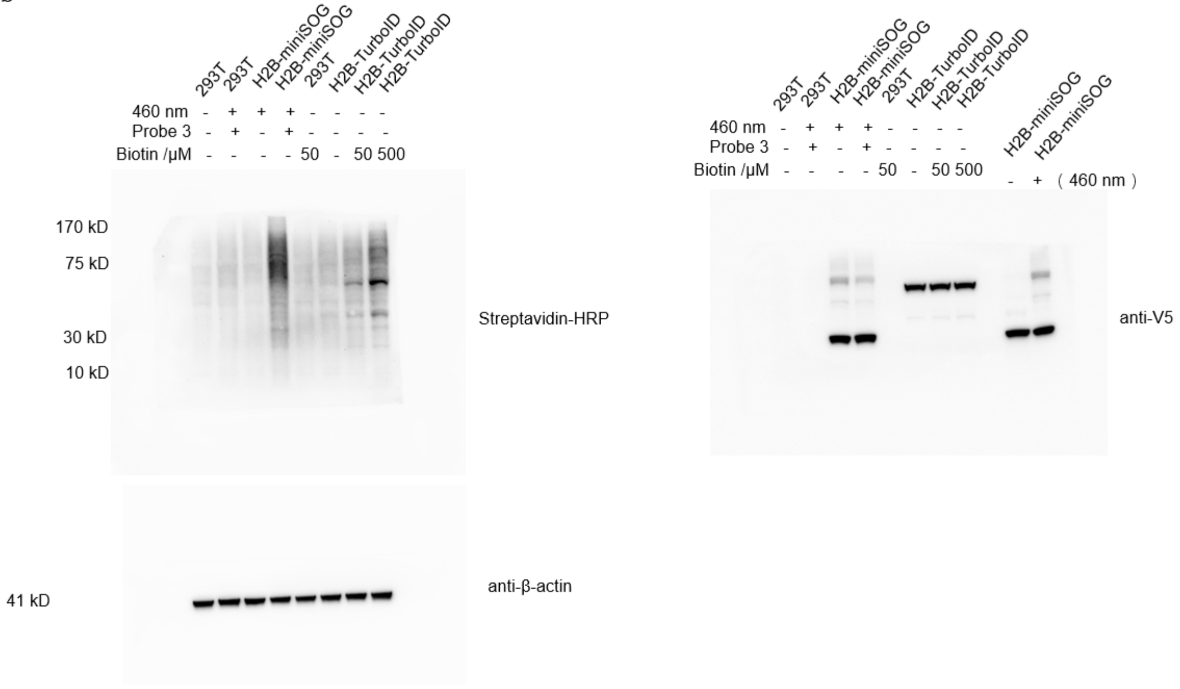

c

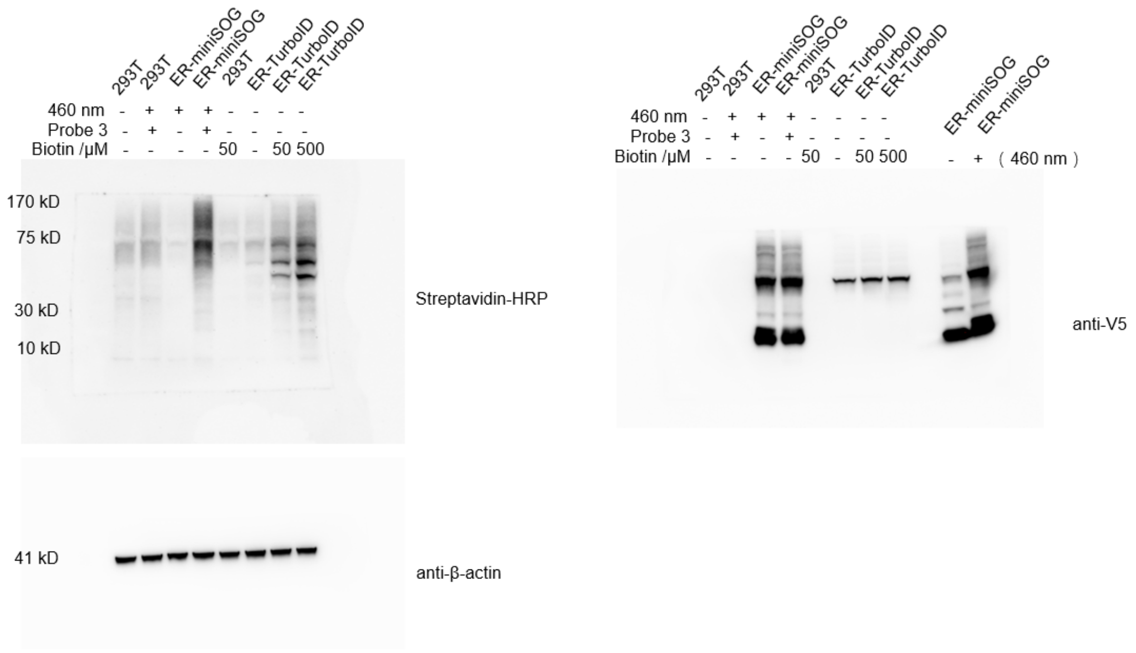

Supplementary Figure 10

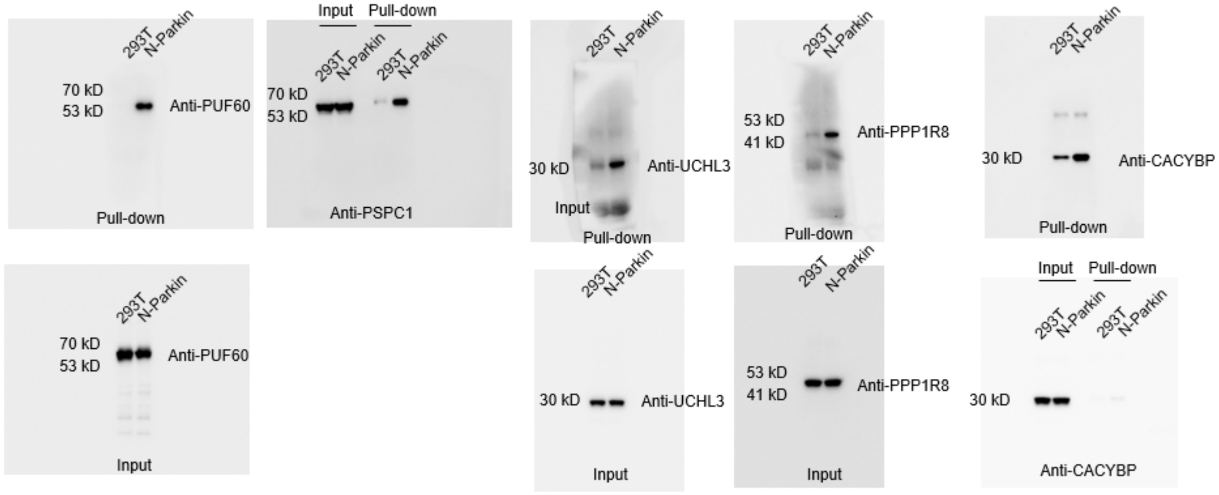

Supplementary Figure 11

a

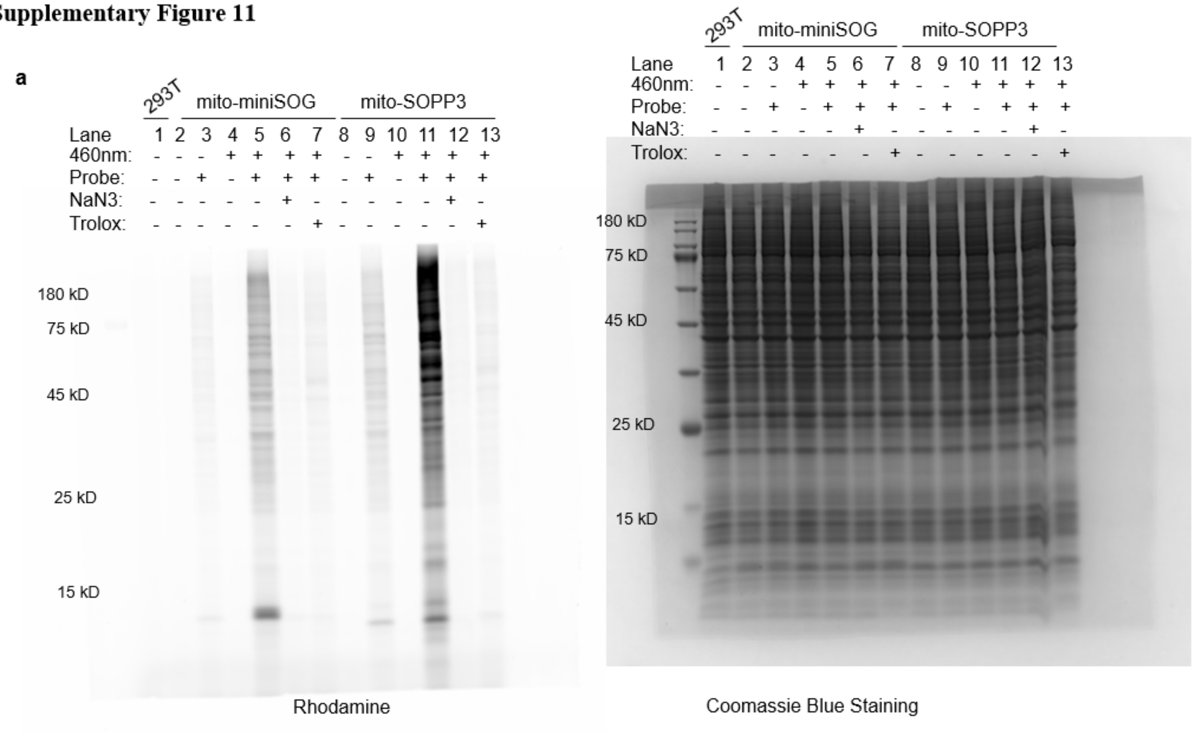

References:

1. O'Shea JP, Chou MF, Quader SA, Ryan JK, Church GM, Schwartz D. pLogo: a probabilistic approach to visualizing sequence motifs. *Nat Methods* **10**, 1211-1212 (2013).

2. Nakane K, *et al*. Proximity Histidine Labeling by Umpolung Strategy Using Singlet Oxygen. *J Am Chem Soc* **143**, 7726-7731 (2021).

3. Mendez-Hurtado J, Lopez R, Suarez D, Menendez MI. Theoretical study of the oxidation of histidine by singlet oxygen. *Chem Eur J* **18**, 8437-8447 (2012).

4. Ziegler DM. Flavin-containing monooxygenases: enzymes adapted for multisubstrate specificity. *Trends Pharmacol Sci* **11**, 321-324 (1990).

5. Grassi L, Cabrele C. Susceptibility of protein therapeutics to spontaneous chemical modifications by oxidation, cyclization, and elimination reactions. *Amino Acids* **51**, 1409-1431 (2019).

6. Pattison DI, Rahmanto AS, Davies MJ. Photo-oxidation of proteins. *Photochem Photobiol Sci* **11**, 38-53 (2012).
