# Supplementary material for: Spatiotemporal-resolved protein networks profiling with photoactivation dependent proximity labeling
Source: Nat Commun. 2022 Aug 20;13:4906. doi: 10.1038/s41467-022-32689-z (PMC9392063; doi:10.1038/s41467-022-32689-z)
Supplement: Supplementary file 3 — Description of Additional Supplementary Files [file 41467_2022_32689_MOESM3_ESM.pdf]

Description of Additional Supplementary Files

File Name: Supplementary Data 1
Description: Open search result of TOP-ABPP workflow with unbiased open search enabled by the MSFragger-based FragPipe computational platform. A summary of the modifications as well as the peptide sequences with “+229” and “+247” modifications on histidine were shown. The relative solvent accessibility (RSA) values were combined together with other peptide-related parameters.

File Name: Supplementary Data 2
Description: Label-free quantification results of subcellular proteomics profiling in mitochondria, ER, nucleus and nuclear lamins by PDPL. Nucleus proteome were profiled by H2B or 3xNLS constructs. Singleton proteins which were only identified in miniSOG expression cells at least twice were included and the corresponding missing values in control cells were replaced from normal distribution by Persues.

File Name: Supplementary Data 3
Description: Label-free quantification results of BRD4-interacting proteins by different illumination time points (2 min, 5 min, 10 min, 20 min) to regulate the labeling radius. Singleton proteins which were only identified in miniSOG-BRD4 expression cells at least twice were included and the corresponding missing values in control cells were replaced from normal distribution by Persues.

File Name: Supplementary Data 4
Description: Label-free quantification results of Parkin-interacting proteins using N-terminal or C-terminal miniSOG constructs. Singleton proteins which were only identified in miniSOG expression cells at least twice were included and the corresponding missing values in control cells were replaced from normal distribution by Persues.
