# Supplementary material for: Spatiotemporal-resolved protein networks profiling with photoactivation dependent proximity labeling
Source: Nat Commun. 2022 Aug 20;13:4906. doi: 10.1038/s41467-022-32689-z (PMC9392063; doi:10.1038/s41467-022-32689-z)
Supplement: Supplementary file 8 — Reporting Summary [file 41467_2022_32689_MOESM8_ESM.pdf]

nature portfolio

Corresponding author(s): Gang Li

Last updated by author(s): Jul 22, 2022

# Reporting Summary

Nature Portfolio wishes to improve the reproducibility of the work that we publish. This form provides structure for consistency and transparency in reporting. For further information on Nature Portfolio policies, see our Editorial Policies and the Editorial Policy Checklist.

## Statistics

For all statistical analyses, confirm that the following items are present in the figure legend, table legend, main text, or Methods section.

| n/a | Confirmed | |
|---|---|---|
| ☐ | ☒ | The exact sample size (*n*) for each experimental group/condition, given as a discrete number and unit of measurement |
| ☐ | ☒ | A statement on whether measurements were taken from distinct samples or whether the same sample was measured repeatedly |
| ☐ | ☒ | The statistical test(s) used AND whether they are one- or two-sided<br>*Only common tests should be described solely by name; describe more complex techniques in the Methods section.* |
| ☒ | ☐ | A description of all covariates tested |
| ☐ | ☒ | A description of any assumptions or corrections, such as tests of normality and adjustment for multiple comparisons |
| ☐ | ☒ | A full description of the statistical parameters including central tendency (e.g. means) or other basic estimates (e.g. regression coefficient) AND variation (e.g. standard deviation) or associated estimates of uncertainty (e.g. confidence intervals) |
| ☐ | ☒ | For null hypothesis testing, the test statistic (e.g. *F*, *t*, *r*) with confidence intervals, effect sizes, degrees of freedom and *P* value noted<br>*Give P values as exact values whenever suitable.* |
| ☒ | ☐ | For Bayesian analysis, information on the choice of priors and Markov chain Monte Carlo settings |
| ☒ | ☐ | For hierarchical and complex designs, identification of the appropriate level for tests and full reporting of outcomes |
| ☒ | ☐ | Estimates of effect sizes (e.g. Cohen's *d*, Pearson's *r*), indicating how they were calculated |

*Our web collection on statistics for biologists contains articles on many of the points above.*

## Software and code

Policy information about availability of computer code

| | |
|---|---|
| Data collection | Mass spectra for PDPL proteomics were acquired using a Thermo Orbitrap Fusion Lumos Tribrid mass spectrometer with the vendor provided Tune and Xcalibur 4.3 software. Microscopy images were acquired on a Zeiss LSM900Airyscan2 confocal microscope with software ZNE 3.5. Gel imaging and western blots were acquired on a Bio-rad Chemidoc MP Touch imaging system with Image Lab Touch Software. The LC-MS of the synthetic peptide was acquired on a Waters Synapt XS Ion Mobility Time-of-Flight mass spectrometer with the vendor provided MassLynx Spectrum analysis software. |
| Data analysis | Mass spectra containing raw files were processed with either Proteome Discoverer 2.5 using SEQUEST search engine or fragpipe 15.0 using Crystal-C and MSfragger. Protein subcellular localization analysis was enabled by Gene Ontology (GO) analysis from DAVID Bioinformatics Resources. Volcano plots were acquired from Perseus version 1.6.15.0. Protein interaction analysis was performed using GO analysis as well as the String database. Confocal images and gel images were processed using ImageJ version 1.53s software. The absolute SASA of each residue was computed using the FreeSASA program (Mitternacht S. F1000Res, 2016, 5, 189). |

For manuscripts utilizing custom algorithms or software that are central to the research but not yet described in published literature, software must be made available to editors and reviewers. We strongly encourage code deposition in a community repository (e.g. GitHub). See the Nature Portfolio guidelines for submitting code & software for further information.

## Data

Policy information about availability of data

All manuscripts must include a data availability statement. This statement should provide the following information, where applicable:

- Accession codes, unique identifiers, or web links for publicly available datasets
- A description of any restrictions on data availability
- For clinical datasets or third party data, please ensure that the statement adheres to our policy

The mass spectrometry raw data in this study are deposited to the ProteomeXchange Consortium (http://proteomecentral.proteomexchange.org) via the iProX partner repository with the dataset identifier PXD034811. SwissProt-reviewed human protein databases were downloaded from Uniprot (https://www.uniprot.org/). Source data and results are provided with this paper. Protein subcellular localization analysis was enabled by Gene Ontology (GO) analysis from DAVID Bioinformatics Resources, MitoCarta 3.0, and database collected and published in Nature Biotechnology paper by Prof. Alice Ting in 2018.

# Field-specific reporting

Please select the one below that is the best fit for your research. If you are not sure, read the appropriate sections before making your selection.

☒ Life sciences ☐ Behavioural & social sciences ☐ Ecological, evolutionary & environmental sciences

For a reference copy of the document with all sections, see nature.com/documents/nr-reporting-summary-flat.pdf

# Life sciences study design

All studies must disclose on these points even when the disclosure is negative.

| | |
|---|---|
| Sample size | For experiments including mass spectrometry, sample size was not predetermined by statistical methods for relative quantitative mass spectrometry experiments. However protein quantities were modeled based on the intensity of at least two peptides using Tukey's median polish method ensuring robust label-free quantification that allows for statistical testing with sample size at least n=3 that is commonly employed for quantitative proteomics investigations (Bantscheff M, et al. Analytical and Bioanalytical Chemistry, 2007, 389, 1017-1031.). For gel analysis and confocal imaging, three biological replicates were performed based on the previously established workflow. |
| Data exclusions | Peptide identifications mapping to decoy or contamination proteins were excluded from further analysis. Peptides were filtered based on the MS/MS identification score calculated in the software to ensure a false discovery rate of < 1 %. To enable the statistical analysis in the Volcano plot, proteins were removed to retain minimally two biological replicates per condition. |
| Replication | Overall, the PDPL proteomics was performed in three biological replicates for label free quantification. For chemical probe optimization, two biological replicates were employed. For the experiment to validate the necessity of PDPL components, three biological replicates were performed. For confocal imaging, western blot, as well as LC-MS, experiments were repeated at least twice with similar results. All attempts at replications were successful and one representative result was shown in the study. |
| Randomization | This study consists of multiple PDPL experiments with a relatively smaller number of the sample (n < 6) that were sequentially acquired by LC-MS/MS to minimize the technical variation for label free quantification, thereby obviating the need for randomization. |
| Blinding | Investigators were not blinded to allocation of biological samples during data collection and/or analysis because the mass spectrometry experiments are of technical nature and not prone to a potential observer bias. |

# Reporting for specific materials, systems and methods

We require information from authors about some types of materials, experimental systems and methods used in many studies. Here, indicate whether each material, system or method listed is relevant to your study. If you are not sure if a list item applies to your research, read the appropriate section before selecting a response.

### Materials & experimental systems

| n/a | Involved in the study | |
|---|---|---|
| ☐ | ☒ | Antibodies |
| ☐ | ☒ | Eukaryotic cell lines |
| ☒ | ☐ | Palaeontology and archaeology |
| ☒ | ☐ | Animals and other organisms |
| ☒ | ☐ | Human research participants |
| ☒ | ☐ | Clinical data |
| ☒ | ☐ | Dual use research of concern |

### Methods

| n/a | Involved in the study | |
|---|---|---|
| ☒ | ☐ | ChIP-seq |
| ☒ | ☐ | Flow cytometry |
| ☒ | ☐ | MRI-based neuroimaging |

# Antibodies

| | |
|---|---|
| Antibodies used | anti-V5 tag mouse mAb (CST, cat:80076, clone:E9H8O), anti-rabbit secondary antibody, Alexa Fluor 594 conjugate (CST, cat:8889), anti-SFPQ rabbit mAb (CST, cat:71992, clone: E9A7B), anti-FUS rabbit mAb (CST, cat:67840, clone: E3O8I), anti-NSUN2 rabbit pAb (Proteintech, cat:20854-1-AP), anti-his tag mouse mAb (TransGen, cat: HT501-01), anti-Flag mouse mAb (TransGen, cat:HT201), anti-β-actin mouse mAb (TransGen, cat:HC201-01), goat anti-mouse secondary antibody, HRP conjugate (TransGen, cat: HS201-01), goat anti-rabbit secondary antibody, HRP conjugate (TransGen, cat: HS101-01), anti-Hsp60 rabbit mAb (ABclonal, cat: A0564), goat anti-rabbit secondary antibody, Alexa Fluor 488 conjugate (Thermo, cat: A11034), anti-mSin3A rabbit pAb (Abcam, cat: ab3479), anti-calnexin rabbit pAb (Abcam, cat: ab22595), anti-CDK2 rabbit mAb (abclonal, cat: A0094), anti-CTBP1 rabbit mAb (abclonal, cat: A11600), anti-DUT rabbit pAb (abclonal, cat: A2901), anti-PSMC4 rabbit pAb(abclonal, cat: A2505), anti-DNAJB1 rabbit pAb (abclonal, cat: A5504), anti-LaminA/C (CST, cat: 2032), anti-streptavidin, HRP conjugate (Solarbio, cat:SE068). |
| Validation | The antibodies include anti-V5 tag mouse mAb (WB, IP, IF, FC; All species), anti-SFPQ rabbit mAb (WB, IP; Human, Mouse, Rat, Monkey), anti-FUS rabbit mAb (WB, IP, IF; Human, Mouse, Rat), anti-NSUN2 rabbit pAb (WB, IP, IHC, ELISA; Human, Mouse), anti-mSin3A rabbit pAb (WB, IF/ICC, IHC, ChIP; Human, Mouse), anti-CDK2 rabbit mAb (WB, IHC; Human, Mouse, Rat), anti-CTBP1 rabbit mAb (WB,IP; Human, Mouse, Rat), anti-DUT rabbit pAb (WB, IF, IHC; Human, Mouse, Rat), anti-PSMC4 rabbit pAb (WB, IF; Human, Mouse), anti-DNAJB1 rabbit pAb (WB, IF; Human, Mouse, Rat), anti-Flag mouse mAb (WB, IP, IF, ELISA; All species), anti-β-actin mouse mAb (WB, IP, IF, ELISA; Human, Mouse, Rabbit), anti-his tag mouse mAb (WB, IP, IF, ELISA; All species), Streptavidin-HRP (WB, IF/ICC, ELISA; All species), anti-LaminA/C (WB, IHC; Human, Mouse, Rat), goat anti-rabbit secondary antibody (WB, ELISA; Rabbit) and goat anti-mouse secondary antibody (WB, ELISA; Mouse) were used in the study for western blot analysis.<br>The antibodies include anti-V5 tag mouse mAb (WB, IP, IF, FC; All species), anti-Hsp60 rabbit mAb (WB, IF, IHC; Human, Mouse, Rat), anti-calnexin rabbit pAb (WB, IP, IF/ICC; Human, Mouse, Rat), anti-mouse Alexa Fluor 594 (IF, FC; Mouse) and anti-rabbit Alexa Fluor 488 (IF/ICC, FC; Rabbit) were used in the study for fluorescence confocal imaging.<br>The primary antibody of mouse anti-V5 tag mouse mAb (WB, IP, IF, FC; All species) was used in the study for co-immunoprecipitation analysis.<br>The primary antibody of anti-his tag mouse mAb (WB, IP, IF, ELISA; All species) was used in the study for in vitro labeling validation. |

# Eukaryotic cell lines

Policy information about cell lines

| | |
|---|---|
| Cell line source(s) | HEK293T cell line was originally purchased from ATCC (CRL-3216). The other stable cell lines used in this study were all generated from HEK293T using lentivirus method. |
| Authentication | In the context of this study, no authentication of HEK293T cell lines was applied. |
| Mycoplasma contamination | All cell lines were tested negative for Mycoplasma contamination. |
| Commonly misidentified lines<br>(See ICLAC register) | No commonly misidentified cell lines were used in this study. |
